# Cardiovascular syphilis-associated acute myocardial infarction: A case report

**DOI:** 10.1097/MD.0000000000024788

**Published:** 2021-02-19

**Authors:** Xiangdong Li, Xue Wang, Zhiyuan Wang, Beibei Du, Cuiying Mao, Heyu Meng, Fanbo Meng, Ping Yang

**Affiliations:** a Department Of Cardiology; b Department of Ultrasound, China-Japan Union Hospital of JiLin University, Changchun, Jilin, China.

**Keywords:** acute myocardial infarction, case report, percutaneous coronary intervention, syphilitic aortitis

## Abstract

**Rationale:**

In recent decades, the incidence of advanced syphilis has declined due to early recognition and the application of effective antibiotics. Advanced syphilis often manifests in the cardiovascular system as simple aortitis, aortic valve insufficiency, coronary artery stenosis or obstruction, Aortic aneurysm and mucinous myocarditis. In most case reports on the subject, acute myocardial infarction caused by syphilis was reported to be due to aortic valve insufficiency and coronary stenosis as a result of the involvement of the aorta.

**Patient concerns:**

The patient was a 48-year-old woman. She was admitted to our hospital because of intermittent upper abdominal pain with chest tightness for 3 hours. The patient reported a past syphilis infection, when she was hospitalized for hysteromyoma surgery four years ago, and had no related treatment.

**Diagnosis:**

According to the characteristics of coronary angiography and results of lab tests and echocardiography, she was finally diagnosed with myocardial infarction associated with syphilis.

**Interventions:**

At the first diagnosis of syphilis, the patient did not received antibiotics treatment. After the diagnosis of myocardial infarction, she received the percutaneous coronary intervention (PCI) operation assisted by extracorporeal membrane oxygenation (ECMO) technology, successfully got drug -eluted stents in right coronary artery ostium and left main ostium. Then the patient received penicillin to treat the syphilis infection.

**Outcomes:**

After coronary revascularization, the cardiac function of the patients was gradually improved, and the left ventricular ejection fraction was gradually improved after combined with optimized drug therapy.

**Lessons:**

The cardiovascular system is often involved in the stages of advanced syphilis with severe complications like myocardial infarction. Standard treatment should be given as soon as syphilis is diagnosis. For stenosis of coronary ostium, the PCI assisted by ECMO technology did not only ensure the effectiveness of the treatment, but also reduce the surgical risk of the patient. This case indicated the effectiveness of ECMO-assisted PCI, and thus may provide a reference for future patient treatment.

## 1 Introduction

Syphilis is a sexually transmitted disease, which has phases of early syphilis and late syphilis according to the course of the disease. In recent decades, the incidence of advanced syphilis has decreased due to early identification and the use of effective antibiotics.^[1]^ However, in recent years, the incidence of syphilis has risen again, and the World Health Organization estimates that there are 12 million new cases of syphilis each year.^[2]^ The cardiovascular system is often involved in the stages of advanced syphilis, with an incidence of up to 10%, which occurs more than 10–30 years after initial infection. Cardiovascular syphilis manifests as simple aortitis, aortic valve insufficiency, coronary artery stenosis or obstruction, aortic aneurysm and mucinous myocarditis^[3–5]^; aortic root aneurysms and aortic valve insufficiency caused by syphilis are common manifestations of syphilis and cardiovascular disease.^[6]^ In rare cases, syphilis can cause coronary artery stenosis associated with thickening of the aortic wall.^[5]^ Among these diseases, acute myocardial infarction (AMI) caused by syphilis is a critical illness among syphilitic heart diseases, due to the aortic valve insufficiency and acute coronary stenosis occurring as a result of aortic involvement. We report a case in which the right coronary ostium is severely stenosed, and the left main coronary ostium is completely occluded without obvious aortic involvement.

## 2 Case presentation

**Figure 1:**
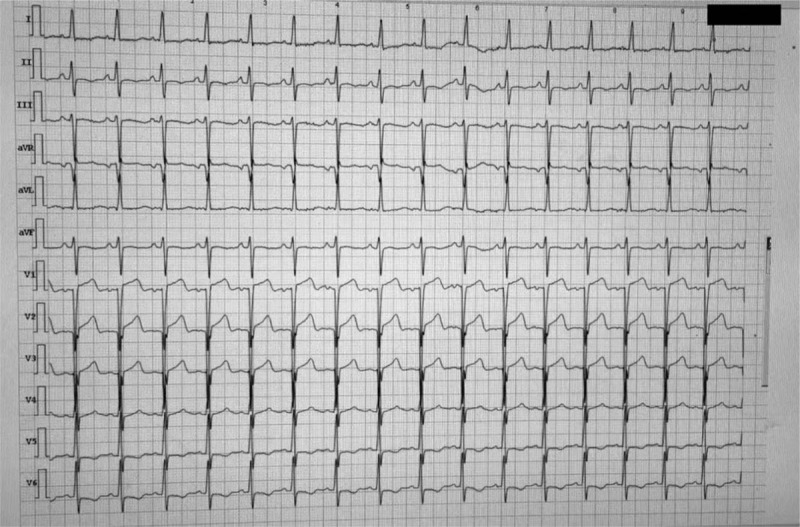
Pathological Q waves in leads V1-V3.

**Figure 2:**
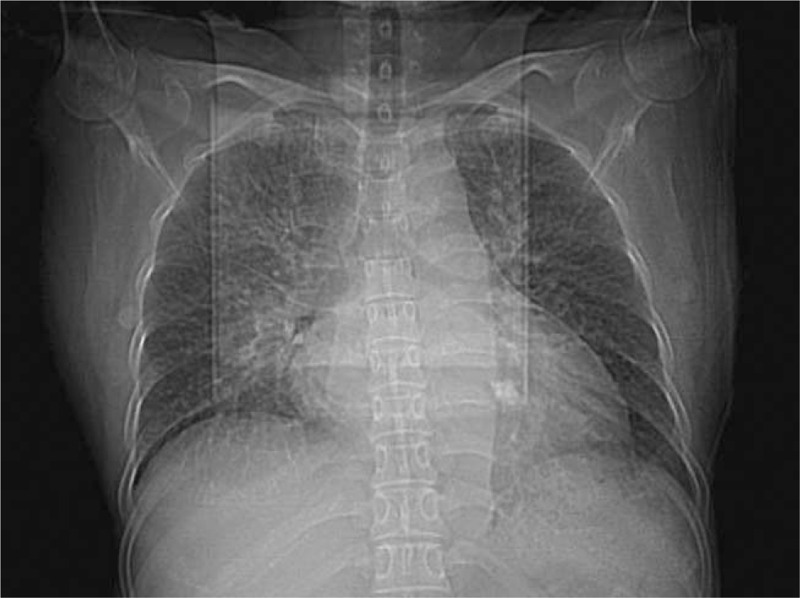
Chest radiograph: Cardiothoracic ratio of 0.69.

**Figure 3:**
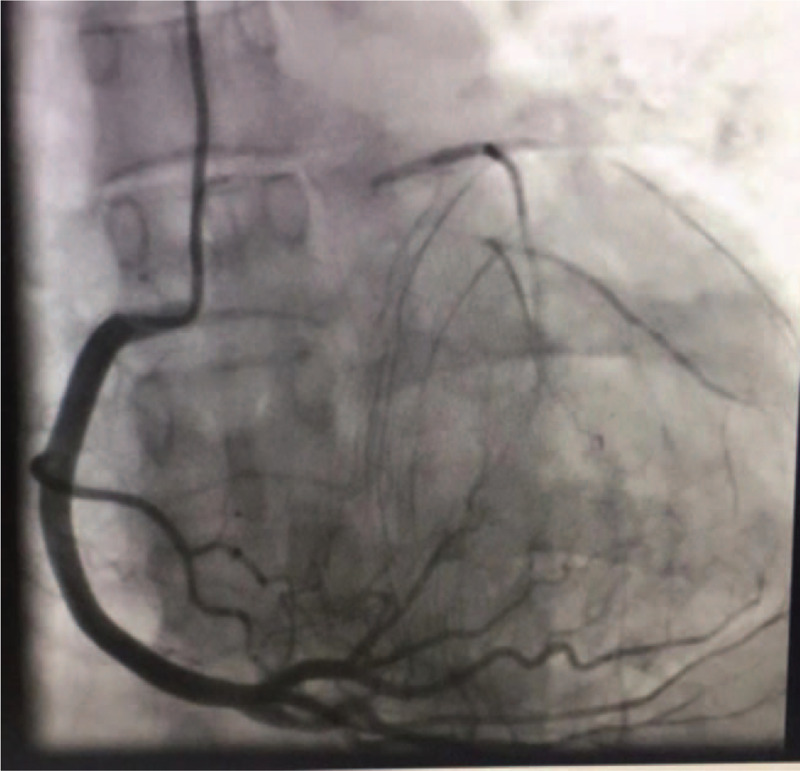
The right coronary artery forms side branches to the left coronary artery (Rentrop score: 3 points).

The patient was a 48-year-old woman. She was admitted to our hospital because of “intermittent upper abdominal pain with chest tightness for 3 hours”. The patient reported a past syphilis infection, when she was hospitalized for hysteromyoma surgery 4 years ago. At that time, the rapid plasma reagin (RPR) antibody test results were positive, with a titer of 1:16. The ECG results were normal. The patient was not given antibiotics to treated the syphilis afterwards. She had no history of hypertension and diabetes, and no family history of cardiovascular disease. The physical examination at admission indicated a heart rate of 110 beats/minute and a blood pressure of 106/75 mm Hg. No significant rales were identified in both lungs; however, the apex was enlarged in the lower left region. Heart sounds were low and blunt. Grade 3/6 blister murmurs could be heard at the apex during systole. There was no obvious upper abdominal pain, and no rebound pain and muscle tension. The liver and spleen were not palpable under the ribs and a negative Murphy's sign was observed. There was no obvious edema in both lower extremities. Mild dyslipidemia (total cholesterol: 6.3 mmol/L and low density lipoprotein cholesterol (LDL-C): 4.21 mmol/L) was also indicated. Troponin: Troponin I levels were at 2.27 ng/dL, and creatinine kinase-myocardial band levels were at 27.6 U/L. Immunization routines suggested the presence of syphilis antibodies (positive test results), and the syphilis RPR titer was 1: 4. An electrocardiogram (ECG) conducted revealed pathological Q waves in leads V1-V3 (Fig. 1). The echocardiogram revealed a staged abnormality in left ventricular wall motion, an increased left atrium and left ventricle, and decreased aortic elasticity. It also indicated possible mild pulmonary hypertension, severe mitral insufficiency (reflux area 9.3 cm^2^), mild mitral and aortic regurgitation, left ventricular diastolic dysfunction (Grade III), reduced left ventricular systolic function (ejection fraction (EF): 29%), and tachycardia. The chest radiograph displayed a cardiothoracic ratio of 0.69 (Fig. 2). As the patient's specific time of onset was unclear, heart failure symptoms had already occurred, and the ejection fraction had decreased significantly. No emergency intervention treatment was given. Conservative treatment for the correction of cardiac function, antiplatelets, statins, beta blockers, Angiotensin converting enzyme inhibitors, and spironolactone were administered. She was not given the antibiotics to treat the syphilis. After 10 days of drug treatment, the patient showed no obvious signs of heart failure. Reexamination of the echocardiogram showed the weakening of left ventricular phase movement, enlargement of the left ventricle, minimal pericardial effusion, mitral valve regurgitation, and mild aortic valve reflux. The measured value of left ventricular systolic function decreased (EF: 29.8%). We also performed a coronary angiography; the results showed that the left main artery was occluded at the ostium, and the right coronary artery ostium was severely stenosis. The right coronary artery forms side branches to the left coronary artery (Rentrop score: 3 points) (Fig. 3). As the patient mainly presented with an involvement of blood vessels at the opening of the coronary artery, arteritis and syphilis damage were not excluded. We reviewed the ultrasound of the subclavian artery and the ultrasound of the carotid artery. There were no obvious plaques and no involvement at the opening. The anti-nuclear antibody test series revealed no obvious abnormalities.

As the only observable risk factor for cardiovascular disease in the patient was a slight increase in LDL-C with no other risk factor detected, the case profile was deemed inconsistent with the profile associated with a diagnosis of severe coronary artery disease. We considered that the coronary stenosis of the patient may have arisen from coronary vasculitis caused by syphilis; the aorta had no obvious involvement.

**Figure 4:**
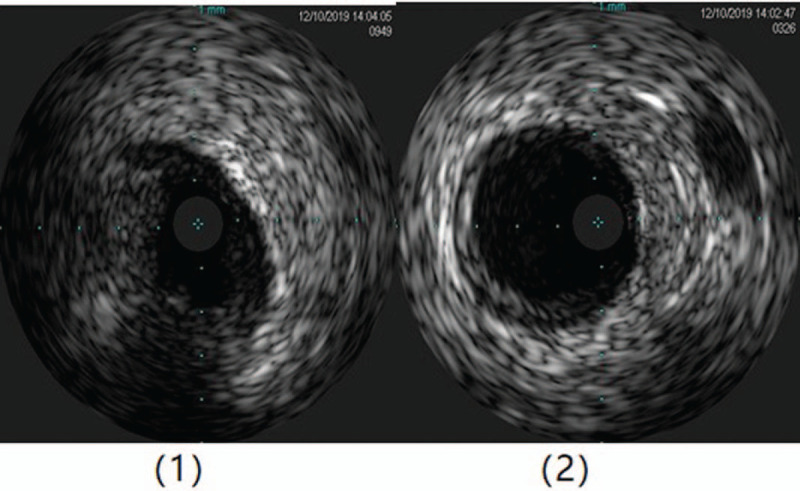
(1) The ostium of the right coronary artery is severely stenosis, the media is unclear and mainly consists of fibrous plaques. (2) There is no obvious plaque beyond the coronary artery ostium.

**Figure 5:**
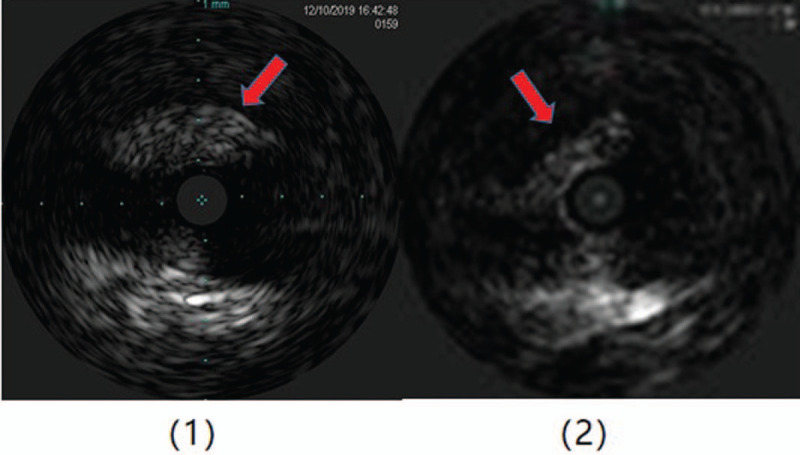
A lump of fibrous tissue was visible between the opening of the left main and aortic sinus, with a size of about 2.75 × 0.68 mm.

For this patient, we believed that a coronary artery bypass graft (CABG) might be effective; however, the patient refused surgical treatment. Due to a poor cardiac function index and the high risk associated with a percutaneous coronary intervention (PCI), we chose extracorporeal membrane oxygenation (ECMO)-assisted PCI therapy as the mode of treatment. During the intraoperative intravenous ultrasound (IVUS) examination, the right coronary artery opening was severely narrowed, the media was unclear, fibrous plaques were observed, and there were no obvious plaques beyond the opening (Fig. 4). A lump of fibrous tissue was visible between the opening of the left trunk and aortic sinus, with a size of about 2.75 x 0.68 mm (Fig. 5), and there was no obvious plaque load on the trunk. We also observed mild plaque load on the anterior descending branch, mainly fiber plaque. A stent was placed at the opening of the right crown. A retro-grade approach was used to open the occlusive lesions at the left coronary artery through collateral circulation of the right coronary atery, and a stent was placed at the opening of the left main. The patient's condition was stable during the operation, and the ECMO device was removed after the operation.

During hospitalization, the patient suffered a ventricular fibrillation due to hypokalemia and cardiac arrest, and recovered after resuscitation. Gastrointestinal bleeding occurred during this period. Administration of aspirin was stopped, and only ticagrelor 90 mg twice a day was given orally. Reexamination of the echocardiogram before discharge indicated a weakening of diffuse left ventricular movement, slight regurgitation of mitral and aortic valves, and a decrease in the value of left ventricular systolic function (EF: 40%). After discharge, the patient was prescribed cilostazol, ticagrelor, atorvastatin, Entresto, metoprolol, and penicillin to treat the syphilis infection. Two months later, there was no obvious discomfort, and there was no obvious abnormality on the electrocardiogram. The echocardiogram displayed a left ventricular inner diameter of 50.6 mm, an EF of 44%, segmental movement abnormalities in the left ventricle wall, the anterior wall and anterior partition wall, and a reduction in the amplitude of left ventricular side wall motion. It was also observed that the left ventricle was relatively enlarged and the apex was rounded, in addition to mild mitral regurgitation, mild aortic insufficiency, reduced left ventricular systolic function, and reduced left ventricular diastolic function. Blood lipid levels were indicated by the total cholesterol level at 4.6 mmol/L and LDL-C levels at 2.9 mmol/L.

## 3 Discussion

Cardiovascular syphilis is the third stage of syphilis infection and falls in the category of advanced syphilis. About 10% of untreated patients develop the disease in 10–30 years after the initial infection,^[4]^ which mainly manifests as asymptomatic aortitis, aortic valve insufficiency, coronary artery stenosis, aortic aneurysm, and mucinous myocarditis.^[3]^ Coronary stenosis caused by syphilis can be associated with the thickening of the aortic wall; about 26% of patients with arteritis have coronary artery involvement.^[7]^ In rare cases, this coronary artery disease can lead to AMI.^[8]^

Coronary stenosis caused by syphilis should be considered an aortic disease.^[9]^ The pathological feature of syphilitic aortitis is vascular occlusive endometritis accompanied by chronic inflammatory infiltration, ischemic necrosis, and intermediate fibrosis. The longitudinal wrinkling of the aortic wall can be caused by aortic scars.^[10]^ This is different from the pathological results of atherosclerosis. In this case, the right coronary artery formed a good collateral blood supply to the left coronary artery. The patient had no previous history of angina, indicating that the occlusion of the left coronary artery formed slowly. The pathological process of this patient is completely different from most of acute myocardial infarction cases which caused by the rupture of atherosclerotic plaque. The results of the IVUS also do not support the possibility of a diagnosis of spontaneous coronary artery dissection.

Syphilis aortitis, and even iatrogenic stenosis may cause coronary artery stenosis, the incidence of which varies between 0.13% and 2.7% among those with coronary heart disease.^[3]^ In patients with no personal history, family history and risk factors of coronary atherosclerosis, patients with AMI, especially those with coronary ostium lesions in the coronary angiography, and patients without distal coronary artery disease, should always be alert for arteritis-related diseases. At present, the true incidence of syphilis aortitis is unclear, but a study with 100 cases who underwent clinical pathological autopsy showed that only 17% of patients were clinically diagnosed with syphilis aortitis.^[5]^ In this case report, the patient had definitely been diagnosed with syphilis infection 4 years ago, with a RPR titer 1:16 and had not been treated systematically, and the syphilis was still active now with a RPR titer 1:4, which help us with a clear diagnosis and treatment clue. The patient has the following characteristics: there are no high-risk factors for coronary heart disease, arterial ultrasound excludes the possibility of aortic arteritis, IVUS results do not support the diagnosis of spontaneous coronary dissection, and coronary angiography indicates that only the coronary ostium is involved. With persistent syphilis infection, combined with these characteristics, we conclude that aortitis caused by syphilis infection involves the coronary arteries and is the cause of acute myocardial infarction in the patient.

It was considered that elective stenting of left main coronary artery lesions without any protective measures is contraindicated;^[11]^ however, one study showed that in a cohort of patients with unprotected left main coronary artery disease, patients with stents and patients with CABG displayed no significant differences in mortality, composite death endpoints, Q-wave myocardial infarction, or stroke.^[12]^ For AMI caused by cardiovascular syphilis lesions, Mutsuo Tanaka *et al* reported the use of CABG to treat cardiovascular syphilis complicated with aortic valve insufficiency and bilateral coronary artery stenosis caused by AMI.^[13]^ Predescu et al. reported that emergency PCI can be used to treat patients with acute anterior myocardial infarction caused by severe left coronary artery stenosis secondary to syphilitic aortitis.^[5]^ For non-atherosclerotic lesions, percutaneous transluminal coronary angioplasty with stents is a safe and effective replacement for CABG.^[11]^ In our case, the patient only had syphilitic aortitis with accumulated coronary artery openings. The aorta, aortic valve, and other blood vessels showed no obvious arterial inflammatory changes, which is different from what has been reported in other syphilis cardiovascular complications. At first, we thought that CABG might be more effective for this patient. The PCI operation took longer and got higher exposure of radiation. The patient shared 5 hours and 45 minutes during the operation and received a total radiation dose of 13106 uGym^2^. The patient only had coronary stenosis and no obvious aortic involvement. Through various evaluations, we chose elective PCI with the assistance of ECMO as the mode of treatment. Additionally, we have also learned a lesson that for patients with coronary ostium occlusion, we must complete coronary Computerised Tomography Angiography before PCI to clarify the location of the coronary ostium, which can reduce the duration and increase the success rate of PCI.

As persistent syphilis infection may cause restenosis in the stent, long-term follow-up of cardiovascular syphilis patients and subsequent antibiotic treatment as soon as possible are important.

## 4 Conclusion

For patients with AMI without coronary heart disease-related risk factors, we must always be alert for diseases related to arteritis. At present, there are no reports of patients with syphilitic cardiovascular disease that only involve the coronary ostium who were treated with elective PCI. However, in our case report, ECMO-assisted PCI achieved positive therapeutic effects in the patient, and thus may provide a reference for AMI-patient treatment in the future. For the syphilisis, systematic and standard treatment can prevent the occurrence of syphilitic cardiovascular complications. Future follow-up is also essential for the prevention of intrinsic stent stenosis and the monitoring of the occurrence of aortitis and severe aortic insufficiency.

## Author contributions

**Conceptualization:** Ping Yang.

**Investigation:** Ping Yang.

**Project administration:** Ping Yang.

**Resources:** Bei-Bei Du.

**Supervision:** Zhiyuan Wang, Bei-Bei Du, Cuiying Mao, Fanbo Meng, Ping Yang.

**Validation:** Xiangdong li, Zhiyuan Wang.

**Visualization:** xiangdong li, Heyu Meng, Ping Yang.

**Writing – original draft:** Xue Wang, Xiangdong Li.

**Writing – review & editing:** Xiangdong li, Cuiying Mao, Ping Yang.
